# Oral melatonin in high-risk critically ill patients: quality of sedative effect

**DOI:** 10.1186/cc10929

**Published:** 2012-03-20

**Authors:** G Sabbatini, G Mistraletti, B Cerri, S Miori, I Galluccio, M Tozzi, C Villa, M Umbrello, F Fraschini, G Iapichino

**Affiliations:** 1 Università degli Studi di Milano, Milan, Italy

## Introduction

Analgesic/sedative therapy is necessary in ICU patients; however, it presents important side effects. Critically ill patients have altered circadian rhythm, delirium and agitation often requiring additional sedation. The dramatically reduced endogenous blood melatonin level (basal and night peaks) could play a role in this context. We evaluated the effects of oral melatonin administration on the adaptation to critical illness and invasive procedures in high-risk critically ill patients [1] consciously sedated [2].

## Methods

Double-blind RCT between placebo and melatonin (3 mg bid, 8:00 and 12:00 p.m., from third ICU day until discharge). Inclusion: age >18, SAPS II >32, expected mechanical ventilation (MV) >4 days, practicability of the gastroenteric tract. Patients were treated according to local guidelines [2], titrating sedatives to a conscious target (Richmond Agitation Sedation Scale (RASS) = 0) as early as possible. Each day, the physician in charge stated the RASS target; nurses assessed the actual RASS.

## Results

Eighty-two patients enrolled: age 72 (60 to 77), SAPS II 41 (34 to 54), MV length 11 (6 to 22) days. Fifteen pancreatitis, 33 acute lung diseases, 13 acute heart diseases, 21 other. The analgesic/sedative therapy during the first 3 days was not different between groups. Melatonin administration determined early weaning from sedatives and analgesics. The prevalence of conscious sedation (RASS = 0) was higher in the melatonin group (67.9 vs. 60.1%, *P *< 0.01), while deeper levels of sedation (RASS = -3/-4) were lower in the melatonin group (RASS -3: 2.4 vs. 7.7%, *P *< 0.01; RASS -4: 1.9 vs. 4.3%, *P *< 0.01). Melatonin administration caused no oversedation (26.3 vs. 24.2%, *P *= 0.94), while decreased undersedation (18.6% vs. 26.2%, *P *= 0.05). RASS targets were joined more frequently in the melatonin group, even if not significantly (55.1 vs. 49.6%, *P *= 0.12).

## Conclusion

Oral melatonin increased the prevalence of conscious sedation in high-risk critically ill patients; it allowed a better achievement of RASS target, particularly decreasing undersedation episodes.

Clinicaltrial.gov NCT00470821
